# Developmental Scarcity Induces Sex Differences in Social Recognition and CA2 Perineuronal Nets

**DOI:** 10.1523/ENEURO.0199-26.2026

**Published:** 2026-09-18

**Authors:** Renée C. Waters, Isha R. Gore, Casey J. Brown, Aimee L. Skweres-Gilmartin, Aarushi B. Rathaur, Elizabeth Gould

**Affiliations:** Princeton Neuroscience Institute, Princeton University, Princeton, New Jersey 08540

**Keywords:** early-life adversity, hippocampus, perineuronal net, postnatal stress, sex difference, social memory

## Abstract

Early-life adversity (ELA) has a lasting negative impact on social behavior in both humans and rodents. Sex differences exist in functional outcomes of postnatal stress, but underlying mechanisms remain incompletely explored. Using the limited bedding and nesting paradigm, a mouse model of developmental scarcity, we found sex differences in social behavior with adult males showing impaired social recognition and adult females showing intact social recognition with enhanced social novelty preference. Perineuronal nets (PNNs), specialized extracellular matrix structures, are known to inhibit developmental plasticity and are concentrated in the CA2 region of the hippocampus in adulthood. We found higher intensity of CA2 PNNs in adult males, but not adult females, after ELA. Focal degradation of the extracellular matrix in the CA2 region restored social recognition function in adult males previously subjected to early stress, suggesting that the effects of developmental adversity may be improved by targeted intervention in adulthood.

## Significance Statement

Early-life adversity (ELA) alters social behavior and increases the likelihood of developing neuropsychiatric disease in humans. Using a mouse model, we found that social memory is negatively affected by ELA in males but not females. This deficit is accompanied by increased intensity of perineuronal nets, extracellular matrix structures that regulate plasticity, in the CA2 region of the hippocampus in ELA males. Lowering PNNs in the CA2 of adult males exposed to ELA restores social memory, suggesting that intervention in adulthood effectively corrects social dysfunction that arises during development.

## Introduction

In humans, childhood adversity increases susceptibility to several neuropsychiatric diseases in adulthood (Felitti et al., 1998). Among the neuropsychiatric diseases linked to early-life adversity (ELA), several present with deficits in social cognition, including posttraumatic stress disorder and major depressive disorder (Beck et al., 2009; Ladegaard et al., 2014; DiGangi et al., 2017). These conditions affect brain regions that are involved in sociocognitive processes, such as the hippocampus, amygdala, and prefrontal cortex (Tanimizu et al., 2017; Helm et al., 2018; Ressler et al., 2022). Substantial evidence suggests that sex and type of ELA are important variables in determining neuropsychiatric outcomes in response to ELA. For instance, emotional abuse is more commonly linked to alcohol use disorder in women while physical abuse and emotional neglect are more commonly linked with clinical depression and anxiety disorders in men (Prachason et al., 2024). Furthermore, several studies have raised the possibility that ELA can have differential neural consequences depending on sex. For example, emotional abuse is more closely tied to reduced hippocampal volume in males than in females, although the overall risk of psychopathology does not differ between sexes (Samplin et al., 2013).

Independent of neuropsychiatric diagnoses, ELA has been shown to affect social cognition. For example, children subjected to emotional abuse have a low threshold for assuming negative emotions on facial expressions in adulthood, while those subjected to emotional neglect have deficits in facial recognition in adulthood (Iffland and Neuner, 2020). Given that social cognition impairments negatively affect quality of life (Schafer and Schiller, 2018), it is important to identify the neural mechanisms underlying ELA effects on social cognition with the goal of establishing points of intervention in adulthood. Toward this end, numerous studies have used animal models of ELA to explore neural and behavioral outcomes, with the majority focused on nonsocial behavioral outcomes, such as avoidance behavior, stress coping, and nonsocial cognition (Rice et al., 2008; Peña et al., 2017; Bolton et al., 2018; Yajima et al., 2018; Laham et al., 2022; Demaestri et al., 2024). A few studies have shown that the ELA paradigm of limited bedding and nesting (LBN), a putative model of resource scarcity (Demaestri et al., 2022), reduced investigation time of novel conspecifics (Kohl et al., 2015), although another study did not find any significant differences in this task (Shupe and Clinton, 2021). LBN has also been shown to reduce sociability (preference for social over nonsocial stimuli) in male mice (Wendel et al., 2021; Maulik et al., 2023).

The hippocampus, which plays an important role in social memory (Kogan et al., 2000; Trinkler et al., 2009; Viskontas et al., 2009), undergoes considerable postnatal development, making it potentially vulnerable to the effects of early adverse events (Dick et al., 2022). Indeed, studies have shown that ELA reduces hippocampal volume in humans (Dannlowski et al., 2012; Paquola et al., 2016) and diminishes neurogenesis, dendritic arborization, and synaptogenesis in rodents (Mirescu et al., 2004; Monroy et al., 2010; Leslie et al., 2011). The CA2 region of the hippocampus has been referred to as a “social hub” because it integrates social information from multiple afferents to support social recognition (Hitti and Siegelbaum, 2014; Diethorn and Gould, 2023a). Afferents to this region, particularly from the dentate gyrus and supramammillary nucleus, undergo considerable development during the postnatal period (Diethorn and Gould, 2023b). In adulthood, the CA2 receives input from adult-born granule cells (abGCs) of the dentate gyrus, which have been linked to social recognition (Cope et al., 2020; Laham et al., 2024). The CA2 also contains a large concentration of perineuronal nets (PNNs), extracellular matrix structures implicated in plasticity inhibition, which have also been linked to social recognition in adulthood (Cope et al., 2022; Alexander et al., 2025). Furthermore, abGCs and PNNs are sensitive to early-life stress (Mirescu et al., 2004; Catale et al., 2022; Laham and Gould, 2022), raising the possibility that ELA may influence social recognition by altering either or both plastic processes.

Here we show that exposure to LBN impairs social recognition function in adult male, but not female, mice. We found that neither LBN males nor LBN females displayed differences in the number of abGCs in the dentate gyrus. However, LBN males, but not females, had increased PNN intensity, specifically of glycosaminoglycan (GAG) chains, in area CA2. Reduction of CA2 PNNs in adulthood restored social memory function in LBN males, showing that persistent dysfunction arising from ELA can be corrected even after development has ended. These findings show that targeted intervention directed at ELA-specific mechanisms can restore social recognition function in adulthood.

## Materials and Methods

### Animals

All animal procedures were approved by the Princeton University Institutional Animal Care and Use Committee and were in accordance with the National Research Council Guide for the Care and Use of Laboratory Animals. Adult C57BL/6J mice were obtained from Jackson Laboratory and bred in the Princeton Neuroscience Institute. Initial studies used mixed-sex groups; however, some follow-up investigations were male-only because LBN-induced behavioral impairments were not observed in females. All mouse pups were weaned from their dams on postnatal day (P)21 and housed in same-sex groups of 4–5 per cage. Mice were housed in Optimice cages on a reverse 12/12 h light/dark cycle.

### LBN

All pups included in these experiments were cross-fostered on P2 and their dams were randomly assigned to the control-rearing or the LBN group. Cross-fostering, which was used to minimize the concentration of biological littermates within a given rearing condition, has been shown to produce only minimal changes to maternal care in C57 mice (van der Veen et al., 2008) and did not result in pup rejection or mortality in our studies. The LBN paradigm was adapted from previous studies (Rice et al., 2008; Gallo et al., 2019). Control litter cages contained standard bedding and a normal amount of nesting material and were left undisturbed until weaning on P21. LBN pups and their dam were moved from their home cages and placed into a cage with a perforated metal floor, no bedding, and very little nesting material from P4 to P11. Following P11, LBN mice were returned to cages with standard bedding and nesting until weaning on P21.

### Behavioral analysis

The direct social interaction test (DSIT) was used to determine whether adult mice were able to discriminate between a novel and familiar mouse of the same sex and age as previously described (Cope et al., 2022; 2023). The DSIT was conducted in a 12 × 12 inch plexiglass arena with black walls and a clear base under low light (10–15 lux). Prior to the test beginning, mice were acclimated to the behavior testing room for at least 30 min and then habituated to the testing box for 5 min. Following habituations to the room and the testing arena, the mice were subjected to a two-trial test. In Trial 1 (novel), the test mouse was placed in the testing arena with a novel stimulus mouse. The mice were allowed to interact freely for 5 min. After a 24 h delay in their respective home cages, Trial 2 (familiar) began in which the test mouse was placed back in the testing arena with the same, now familiar, mouse for 5 min. The arena was thoroughly cleaned with 70% ethanol between each mouse and trial. Both trials were recorded using a single camera. A trained experimenter, blind to the treatment group, manually scored the time the test mouse spent investigating the stimulus mouse. Social investigation was defined as the test mouse directing its nose toward the stimulus mouse's head, body, or anogenital region within 2 cm, or following or initiating allogrooming. Because healthy adult mice prefer novelty, social recognition is inferred by a decrease in investigation time from the novel trial to the familiar trial (Moy et al., 2004). The discrimination index was calculated as follows: Discrimination Index = [(Novel investigation time minus Familiar investigation time) divided by (Novel investigation time plus Familiar investigation time)].

Because social behavior in females has been linked to sexual receptivity and estrous cycle stage (Sánchez-Andrade and Kendrick, 2011; Chari et al., 2020), female mice were subjected to vaginal smears for 2 weeks prior to behavioral testing, and their performance on the DSIT was compared at stages of sexual receptivity (SR; proestrus and estrus) with stages of sexual nonreceptivity (NR; metestrus and diestrus).

### Surgical manipulations

Adult male mice were deeply anesthetized with isoflurane (2–3%) and placed in a stereotaxic apparatus (David Kopf Instruments) on a temperature-controlled thermal blanket for all surgeries. A microsyringe was used to deliver bilateral infusions of penicillinase (pnase; Sigma-Aldrich, catalog #P0389) or chondroitinase ABC (chABC; Sigma-Aldrich, catalog #C2905), an enzyme known to degrade GAG chains on PNNs (Bradbury et al., 2002; Pizzorusso et al., 2002) to the dorsal CA2 of control and LBN C57 adult males (15 nl per injection at 3 nl/min; AP, −1.82; ML, ±2.15; DV, −1.67). Mice were tested 10 d postinfusion in the DSIT. This postsurgical time point was selected because previous studies showed that at 5 d after CA2 chABC infusion, PNNs remain diminished, and social recognition is impaired, whereas at 10 d after chABC infusion, CA2 PNNs and social recognition are restored in control mice (Cope et al., 2022).

### Histology

Mice were anesthetized with EUTHASOL (Virbac) and transcardially perfused with cold 4% paraformaldehyde (PFA). Brains were postfixed for 48 h in 4% PFA at 4°C followed by an additional 48 h in 30% sucrose at 4°C for cryoprotection before being frozen in tissue freezing medium at −80°C. Hippocampal coronal sections (40 μm) were collected using a cryostat (Leica). Sections were incubated for 1.5 h at room temperature in a PBS solution that contained 0.3% Triton X-100 and 3% normal donkey serum. Sections were then incubated overnight while shaking at 4°C in the blocking solution that contained one or two of the following: mouse anti-3 microtubule-binding domain tau protein (3R-Tau; 1:500, Millipore, catalog #05-803) to label immature neurons, rabbit anti-Purkinje cell protein 4 (PCP4; 1:500, Sigma-Aldrich, catalog #HPA005792) or mouse anti-regulator of G-protein signaling 14 (RGS14; 1:500, Antibodies Incorporated, catalog #75-170) to label the CA2 region, and rabbit anti-aggrecan (ACAN; 1:1,000, Millipore, catalog #AB1031) to label the main neuronal chondroitin sulfate proteoglycan (CSPG) of PNNs or the plant lectin *Wisteria floribunda* agglutinin (WFA; 1:1,000, Sigma-Aldrich, catalog #L1516) to label GAG chains on PNNs.

For 3R-Tau immunohistochemistry, sections were subjected to an antigen retrieval protocol that involved incubation in sodium citrate and citric acid buffer for 30 min at 80°C before the blocking solution. All sections were then incubated for 1.5 h at room temperature in secondary antibody solutions that contained combinations of the following secondaries: donkey anti-rabbit Alexa Fluor 568, donkey anti-mouse Alexa Fluor 488, donkey anti-rabbit Alexa Fluor 488, or Streptavidin 488 (1:500, Abcam). Sections were then counterstained with Hoechst 33342 for 10 min (1:5,000 in PBS, Molecular Probes), mounted onto slides, and coverslipped with VECTASHIELD (Vector Laboratories). Slides were coded until completion of the data analysis.

### Optical intensity measurements

*Z*-stack images of the CA2 were taken using a 40× objective with a 0.5 μm step size through the entire 40 μm tissue section on a Leica SP8 confocal with the LAS X software (version 35.6). The CA2 region was defined by PCP4 or RGS14 labeling, which are specific to the CA2 in the hippocampal CA fields (San Antonio et al., 2014). Collected *z*-stack images of WFA and ACAN were analyzed for optical intensity in Fiji (NIH) as previously described (Cope et al., 2022). For overall intensity values in the CA2 region, the area, mean gray value, and integrated density of the pyramidal cell layer were calculated for each *z*-slice, and the maximum integrated density value for each *z*-stack was taken from three sections per brain. Averages were obtained for each brain, and statistical comparisons were made between groups.

### Cell density measurements

The number of 3R-Tau^+^ cells was manually counted in the dorsal dentate gyrus of the hippocampus on three neuroanatomically matched sections using Fiji (ImageJ). The area measurements were collected using Fiji. The density of 3R-Tau^+^ cells was determined by dividing the total number of positively labeled cells by the volume of the subregion (ROI cross-sectional area multiplied by 40 μm section thickness).

### Statistical analyses

To investigate potential sex differences in behavioral and brain effects of LBN, data from males and females were analyzed together with some additional analyses involving individual sexes. For behavioral analyses, discrimination index and total investigation time datasets were analyzed using two-way ANOVAs (with sex and rearing condition as variables) followed by Tukey post hoc comparisons. Novel and familiar investigation time datasets were analyzed for each sex using two-way repeated-measures ANOVAs with Holm–Sidak post hoc comparisons. For histological analyses, 3R-Tau–labeled cell density, as well as WFA and ACAN intensity across sexes and rearing groups, two-way ANOVAs followed by Tukey post hoc comparisons were used. For WFA intensity analysis between pnase- and chABC-treated mice, data were analyzed using unpaired *t* tests. For behavioral analyses involving chABC manipulations in adulthood, datasets were analyzed using a two-way ANOVA or a repeated-measures three-way ANOVA. Tukey or Holm–Sidak post hoc comparisons were used. *p* < 0.05 was set for statistical significance and *n* = number of pups. GraphPad Prism 9.2.0 was used for statistical analyses and graph preparation.

## Results

### LBN impairs social recognition in adult male mice while enhancing social novelty preference in adult female mice

**Figure 1.**
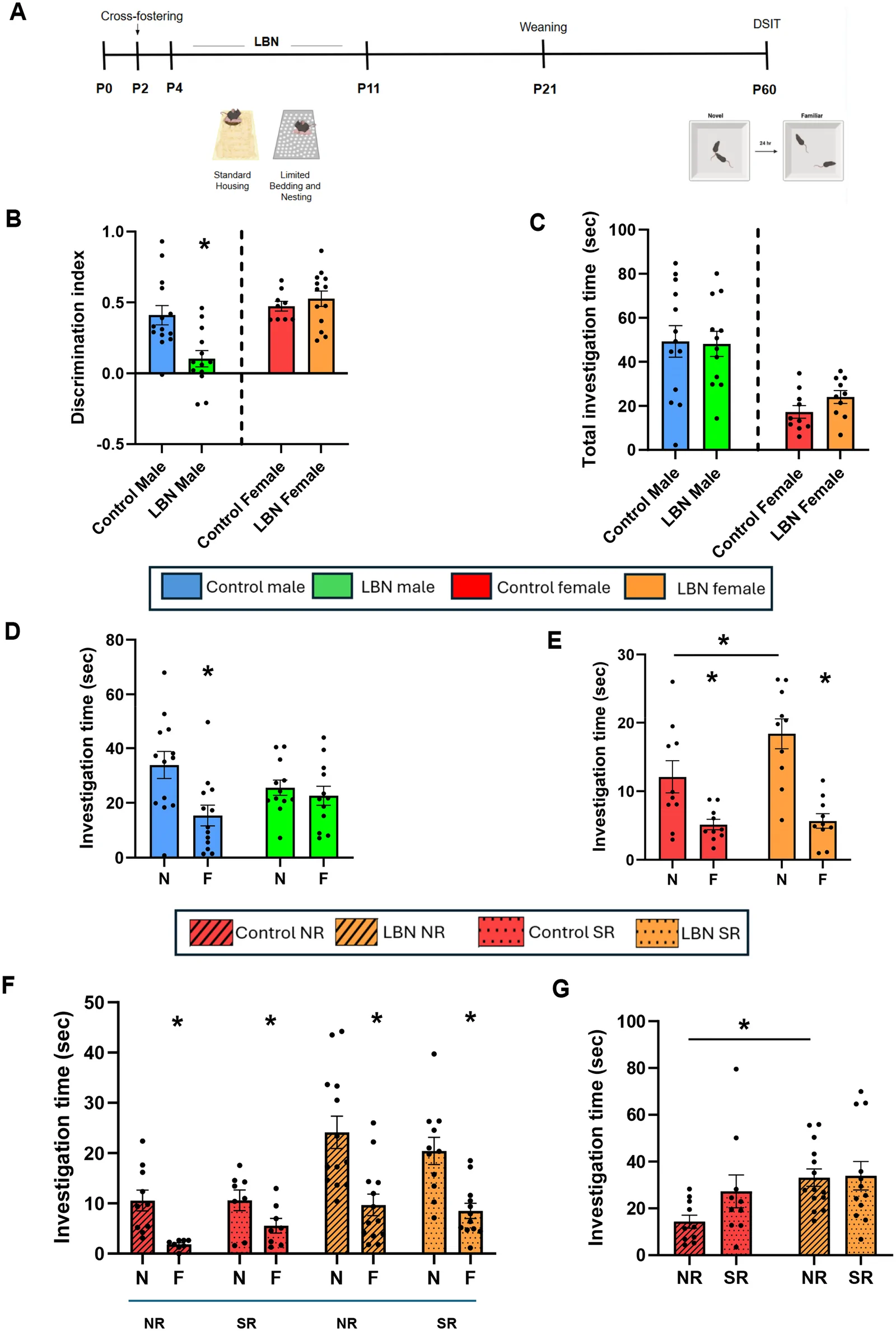
LBN impairs social recognition in adult males, but not adult females. ***A***, Experimental timeline and schematic of LBN paradigm and adult behavioral testing. ***B***, Discrimination indices [(N − F)/(N + F)] showed a sex by rearing condition interaction with LBN males statistically lower than control-reared males, with no statistical differences in this measure between LBN and control-reared females. ***C***, Overall investigation times (N + F) were not different between LBN and control-reared males nor between LBN and control-reared females, although females had lower investigation times than males. ***D***, Control-reared males displayed a significant reduction in investigation time between exposure to the novel mouse in Trial 1 (*N*) to the familiar mouse in Trial 2 (*F*). LBN males showed similar investigation times in Trial 1 and Trial 2. ***E***, Both control-reared and LBN females display a significant reduction in time investigating the novel mouse in Trial 1 to the familiar mouse in Trial 2. LBN females show an increase in novel investigation compared with control-reared females. ***F***, Control-reared and LBN females displayed lower investigation times with familiar than novel social stimuli regardless of whether the mice were tested when sexually receptive or nonreceptive. ***G***, Control-reared females had lower overall investigation times than LBN females when they were nonreceptive, while sexually receptive control-reared and LBN females showed no statistical difference. *n* = 10–13 mice per group; *p < 0.05; N, novel social stimulus; F, familiar social stimulus; NR, nonreceptive; SR, sexually receptive.

Statistical analyses of the direct social interaction behavioral data revealed a significant sex by rearing condition interaction with discrimination indices of control-reared and LBN male and female mice (*F*_(1,45)_ = 9.089, *p* = 0.0042) with significant main effects of sex (*F*_(1,45)_ = 16.63, *p* = 0.0002) and rearing condition (*F*_(1,45)_ = 4.535, *p* = 0.0387). Post hoc comparisons showed that LBN males had significantly lower discrimination indices than control males (*p* = 0.0019), control females (*p* = 0.0008), and LBN females (*p* < 0.0001; Fig. 1*B*), suggesting social recognition impairment. As previously reported (Karlsson et al., 2015; Granza et al., 2023), females had lower overall investigation times than males, but there were no statistically significant interactions or rearing condition effects on this measure (Fig. 1*C*), suggesting that the LBN-induced deficit in social recognition in males was not due to an overall change in social motivation but rather an inability to discriminate between novel and familiar mice. Analyses of individual sex data showed that control-reared male mice had significantly lower investigation times for the familiar mouse than the novel mouse, while LBN male mice showed no significant differences in investigation time across trials (control/LBN × novel/familiar interaction: *F*_(1,23)_ = 6.881, *p* = 0.0152; novel/familiar main effect: *F*_(1,23)_ = 13.09; *p* = 0.0014; novel/familiar post hoc comparisons: control *p* = 0.0003; LBN *p* = 0.7472; Fig. 1*D*). Like control-reared females, LBN females showed a significant decline in investigation time between novel and familiar trials (control/LBN × novel/familiar interaction: *F*_(1,18)_ = 4.643; *p* = 0.0450; novel/familiar main effect: *F*_(1,18)_ = 54.20; *p* < 0.0001; novel/familiar post hoc comparisons: control *p* = 0.0017; LBN *p* = 0.0001; Fig. 1*E*). LBN females had statistically higher investigation time of novel social stimuli (control vs LBN novel: *p* = 0.0146; Fig. 1*E*).

### Stage of estrous does not substantially interact with rearing condition in females

To determine whether stages of estrous/sexual receptivity potentially obscure negative effects of LBN on social recognition in females, we analyzed the data according to whether mice were in estrous stages associated with sexual receptivity (proestrus and estrus) versus sexual nonreceptivity (metestrus and diestrus). Although main effects were observed with rearing conditions (control/LBN: *F*_(1,12)_ = 22.09; *p* = 0.0005) and trial (novel/familiar: *F*_(1,21)_ = 38.38; *p* = 0.0001), no effect of sexual receptivity was observed (*F*_(1,20)_ = 2.111; *p* = 0.1618; Fig. 1*F*). No overall differences in social investigation time (novel + familiar) were detected across stages of receptivity (*F*_(1,20)_ = 2.111; *p* = 0.1618) and LBN females only showed significantly higher investigation times during stages of nonreceptivity (control vs LBN post hoc comparisons: NR *p* = 0.0233; SR *p* = 0.6245; Fig. 1*G*).

### LBN does not affect the number of immature neurons in the dorsal dentate gyrus of males or females

**Figure 2.**
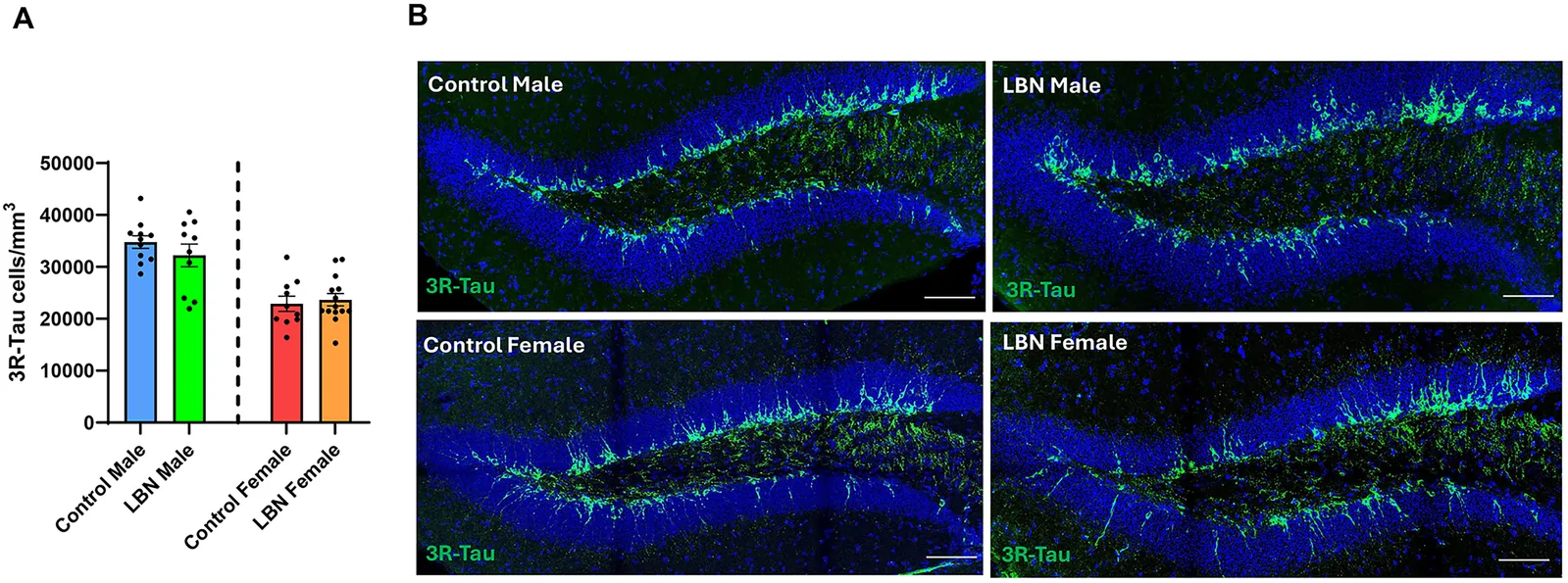
The number of immature neurons in the dorsal dentate gyrus is unaffected in adult male or female LBN mice. ***A***, The number of neurons labeled with 3R-Tau in the dentate gyrus is similar between control-reared and LBN males as well as between control-reared and LBN females. There were no effects of rearing, but females in both groups had lower numbers of immature neurons than males in both groups. ***B***, Representative confocal images of 3R-Tau^+^ cells (green) in the dorsal dentate gyrus of control-reared and LBN adult males and females. Sections were counterstained with the DNA dye Hoechst 33342 (blue). Scale bars, 200 μm. 3R-Tau, 3 microtubule-binding domain tau protein. *n* = 10–14 mice per group.

abGCs are critical for social recognition in healthy mice (Cope et al., 2020), and, through their afferents to area CA2, facilitate social memory retrieval and play an important role in overall CA2 network activity (Laham et al., 2024). Since LBN impaired social recognition in adult males, we hypothesized that a reduction in adult neurogenesis might contribute to this effect, so we investigated whether LBN affects the number of abGCs in males and females. Using 3R-Tau to label immature granule cells in dorsal dentate gyrus (Llorens-Martin et al., 2015), we observed a main effect of sex in 3R-Tau cell density (*F*_(1,41)_ = 45.43, *p* < 0.0001), but no effect of rearing condition (*F*_(1,41)_ = 0.3601, *p* = 0.5517), nor interaction between sex and rearing condition (*F*_(1,41)_ = 1.195, *p* = 0.2807; Fig. 2*A*,*B*). Post hoc comparisons showed that control-reared females had lower densities of 3R-Tau–labeled cells than control-reared males (*p* < 0.0001), and a similar relationship was observed between LBN females and LBN males (*p* = 0.0010). These findings suggest that although sex differences exist in the number of adult-generated immature neurons in the dentate gyrus, if adult neurogenesis plays a role in LBN-induced impairments in social recognition, it does not do so through detectable changes in this measure.

### LBN male, but not female, mice exhibit higher CA2 PNN intensity compared with control-reared mice

Studies have shown that CA2 PNNs are important for the development and maintenance of social recognition abilities in mice (Cope et al., 2022; Diethorn et al., 2023b, 2026), so we investigated the possibility that CA2 PNNs differ between control-reared and LBN mice. Labeling with the plant lectin WFA, which binds to GAG chains on PNNs, showed a significant interaction between sex and rearing condition (*F*_(1,40)_ = 8.311, *p* = 0.0063) with no significant effects of sex (*F*_(1,40)_ = 1.633, *p* = 0.2087) or rearing condition (*F*_(1,40)_ = 1.780, *p* = 0.1898).

**Figure 3.**
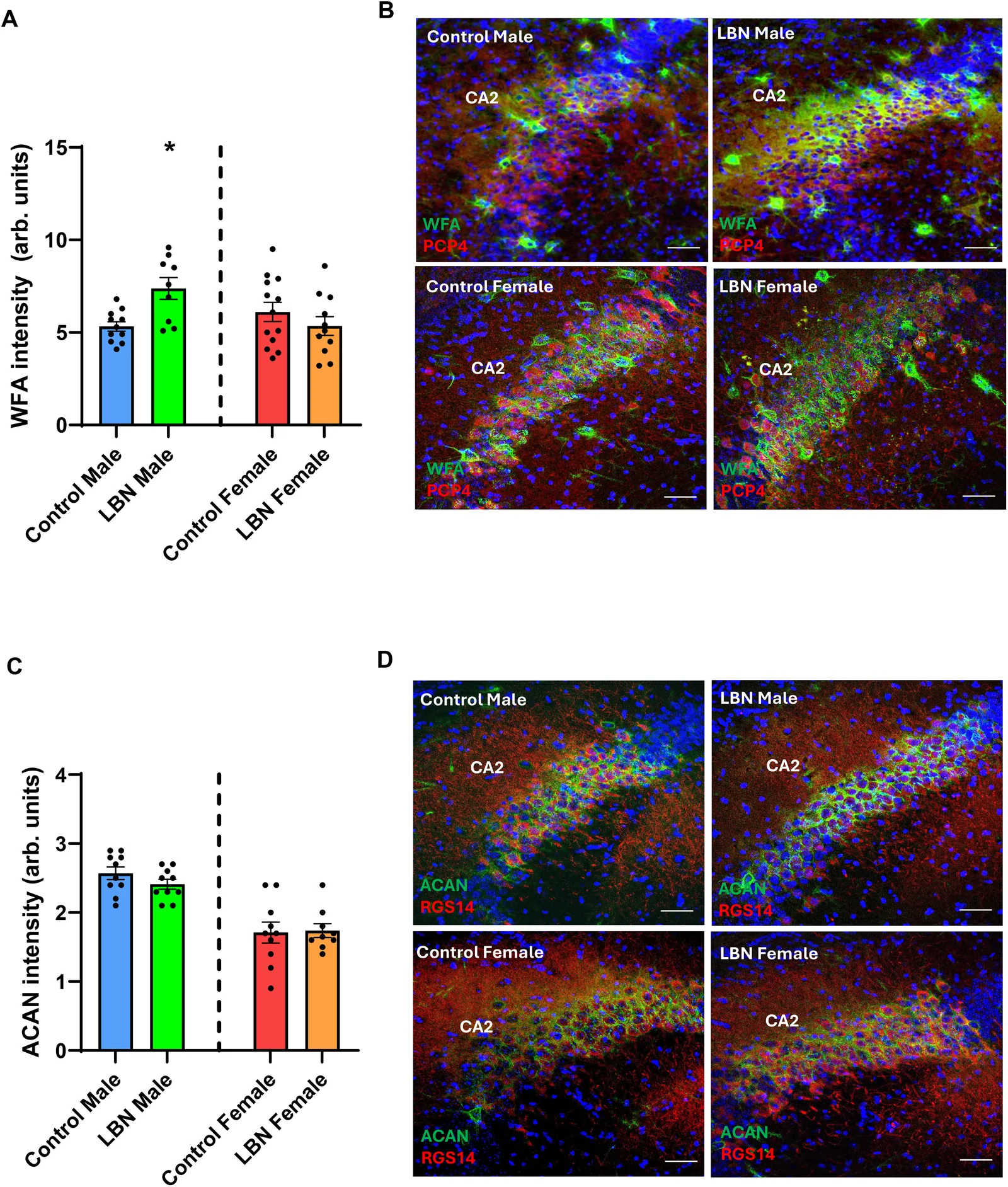
LBN males have higher WFA^+^, but not ACAN^+^, intensity in the CA2 region. ***A***, Analysis of CA2 WFA intensity showed a significant interaction between sex and rearing condition. LBN males had significantly higher intensity of WFA^+^ staining in the pyramidal cell layer of the CA2 compared with control-reared males while LBN females did not show this effect. ***B***, Confocal images of WFA^+^ staining (green) in dorsal CA2 labeled by PCP4 (red) of control-reared and LBN males and females. ***C***, LBN males and females showed no difference in ACAN^+^ intensity in the pyramidal cell layer of the CA2 compared with control-reared males and females, respectively. ***D***, Confocal images of ACAN labeling (green) in dorsal CA2 labeled by RGS14 (red) of control-reared and LBN males and females. Sections were counterstained with the DNA dye Hoechst 33342 (blue). Scale bars, 50 μm; *n* = 9–13 mice per group; *p < 0.05. WFA, *Wisteria floribunda* agglutinin; ACAN, aggrecan; PCP4, Purkinje cell protein-4; RGS14, regulator of G-protein signaling 14.

LBN male mice had >30% higher CA2 WFA intensity than control-reared male mice (*p* = 0.0332), an effect that was not observed in LBN female mice (*p* = 0.6615; Fig. 3*A*,*B*). Despite finding increased WFA intensity in area CA2 of LBN male mice, we found no significant sex by rearing condition interaction (*F*_(1,35)_ = 0.7579, *p* = 0.3899) nor main effect of rearing condition (*F*_(1,35)_ = 0.3652, *p* = 0.5496) in intensity of the main neuronal CSPG ACAN between rearing conditions, although a main effect of sex was observed (*F*_(1,35)_ = 49.80, *p* < 0.0001). These findings suggest that LBN males have altered PNN composition (more GAG chains) as opposed to an overall increase in all PNN components. No LBN-induced differences were observed in the cross-sectional area of the CA2 region in males (control: 35,625 ± 2430; LBN: 36,059 ± 2,614; *t*(20) = 0.1197, *p* = 0.9059) or females (control: 32,930 ± 2665; LBN: 32,772 ± 2,406; *t*(18) = 0.04387, *p* = 0.9655).

### Reducing CA2 PNNs in LBN males restores social recognition

**Figure 4.**
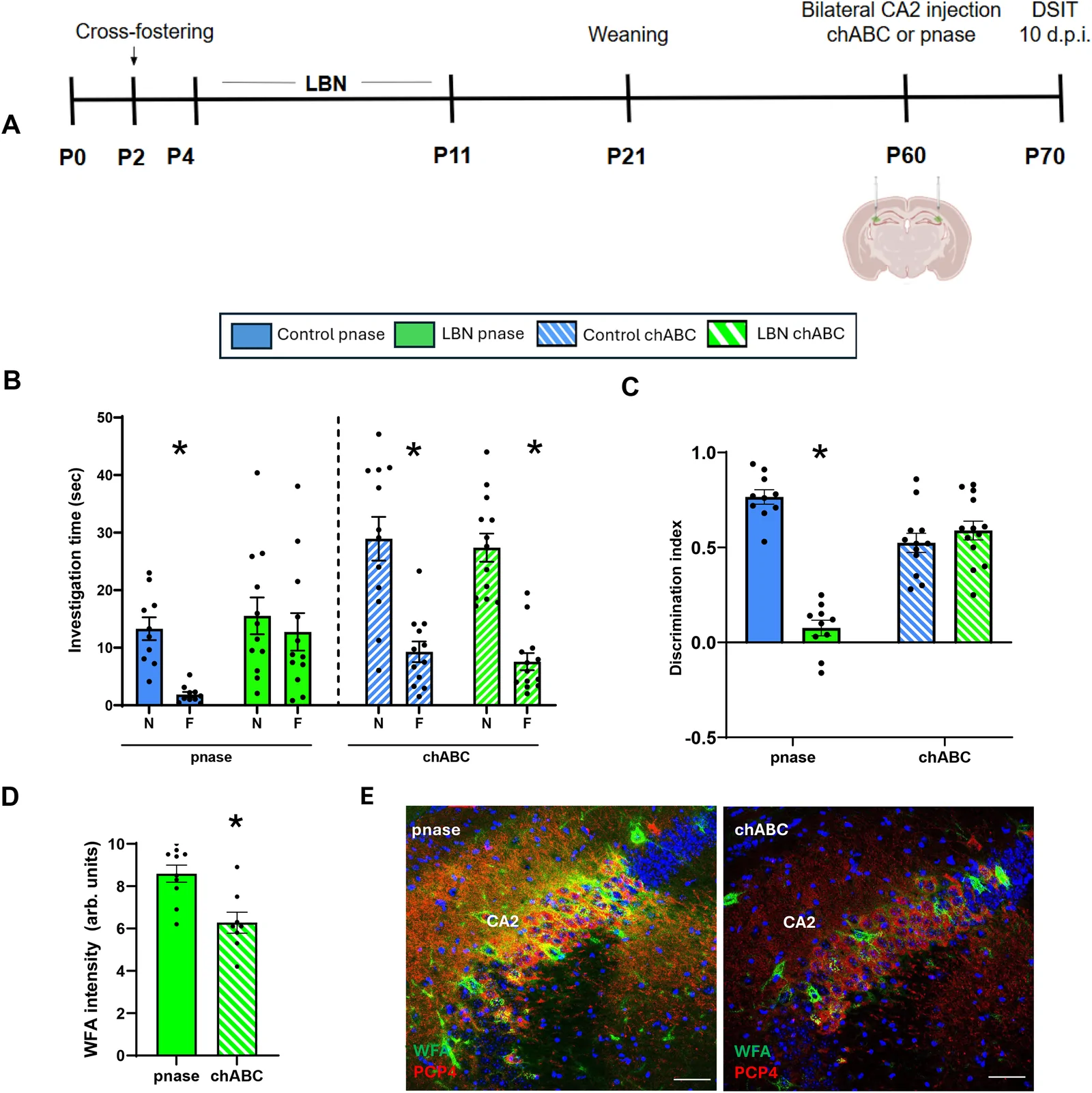
Reducing CA2 PNNs restores social recognition in LBN males. ***A***, Timeline of LBN chABC behavioral experiment. ***B***, Reducing CA2 PNN levels restored social recognition in LBN males. Control pnase, control chABC, and LBN chABC males display a significant reduction in time investigating the novel mouse in Trial 1 to the familiar mouse in Trial 2. LBN pnase males showed similar investigation times in Trial 1 and Trial 2. ***C***, LBN pnase males had significantly lower discrimination indices compared with control pnase, control chABC, and LBN chABC males. ***D***, LBN chABC males had lower WFA intensity in the CA2 than LBN pnase males. ***E***, Confocal images of WFA labeling (green) in dorsal CA2 labeled with PCP4 (red) of LBN pnase and LBN chABC males. Sections were counterstained with the DNA dye Hoechst 33342 (blue). Scale bars, 50 μm; *n* = 11–14 mice per group for behavioral studies, *n* = 8–9 mice per group for histology; *p < 0.05. pnase, penicillinase; chABC, chondroitinase ABC; WFA, *Wisteria floribunda* agglutinin; PCP4, Purkinje cell protein 4; DSIT, direct social interaction test; d.p.i., days postinjection.

Because higher CA2 WFA intensity paralleled social recognition impairment in LBN males, we next investigated whether reducing GAG chains on PNNs with the enzyme chABC (Bradbury et al., 2002; Pizzorusso et al., 2002) in the CA2 would improve social memory. Control-reared and LBN adult males were infused with chABC or the control enzyme pnase in the bilateral CA2 (Fig. 4*A*). Mice were tested on the direct social interaction task 10 d postinjection, a timepoint when WFA-labeled PNNs in the CA2 of control mice have regrown after chABC infusion to control-reared baseline levels, and there is no longer a social recognition deficit (Cope et al., 2022). There was a significant interaction between rearing condition and CA2 infusion treatment in both trial-by-trial (novel, familiar) analysis (control/LBN × chABC/pnase interaction: *F*_(1,39)_ = 4.529, *p* = 0.0397; Fig. 4*B*) and the discrimination index (control/LBN × chABC/pnase interaction *F*_(1,43)_ = 38.85, *p* < 0.0001; Fig. 4*C*). As expected based on previous findings (Cope et al., 2022), we found that 10 d after surgery, control-reared males with pnase and chABC CA2 infusions had typical social recognition abilities, investigating novel mice significantly more than familiar mice (Fig. 4*B,C*). In contrast, LBN males with pnase infusions showed impaired social recognition, investigating novel and familiar mice at similar levels (Fig. 4*B*,*C*). However, 10 d after chABC infusion, LBN males had restored social discrimination abilities comparable to pnase control-reared males (Fig. 4*B*,*C*). LBN mice given pnase had significantly lower discrimination indices compared with all other groups (Fig. 4*C*). After perfusion, the efficacy of chABC to reduce PNNs in LBN males was assessed with WFA labeling. LBN males treated with chABC had significantly lower WFA intensity than LBN males treated with pnase (*t*(14) = 2.171, *p* = 0.0477; Fig. 4*D*,*E*).

## Discussion

We found that LBN impaired social recognition in adult males. In contrast, we found that LBN did not impair social recognition in females but increased novel social investigation. In LBN females, preserved social recognition occurred regardless of sexual receptivity/stage of estrous. Since females in this study were subjected to repeated vaginal sampling to assess estrous stage, it is possible that the additional handling served to mitigate the negative effects of LBN on social recognition in females but not males. However, recent evidence that postpubescent (P45) LBN female mice that had not been handled repeatedly had intact social recognition (Bapat et al., 2026) argues against this possibility. We also found sex differences in measures of hippocampal plasticity, but not all showed a significant sex difference in effect of LBN. While females had fewer immature neurons in the dentate gyrus than males, a sex difference reported previously, we found no differences in this measure in males or females after exposure to LBN. Notably, our analysis of immature granule cells in the adult dentate gyrus was based on a single measure (i.e., the number of 3R-Tau labeled cells), leaving open the possibility that LBN induces changes in the survival of newborn neurons, their dendritic differentiation, and synaptic connections with target sites. Assessing these possibilities should be the focus of future studies. In contrast to the lack of LBN effects on immature granule cells, LBN-reared males had significantly higher PNN intensity in the CA2 compared with controls, while females exposed to LBN showed no differences. Because LBN-induced social memory impairment was associated with atypically high PNNs in adult males, we tested the effects of lowering PNNs in the CA2 region in adulthood and found improved social recognition. These findings suggest that adult intervention may be sufficient to temporarily reverse some negative effects of developmental adversity.

### Reducing CA2 PNNs is associated with restored social recognition in LBN males

Previous studies have shown that the CA2 region plays an important role in social recognition both during development (Laham et al., 2021; Diethorn and Gould, 2023a) and in adulthood (Hitti and Siegelbaum, 2014; Laham et al., 2021; 2024). There is an unusually high concentration of PNNs in the healthy CA2, which surround both inhibitory interneurons, as is typical for other hippocampal subregions, and pyramidal cells (Carstens et al., 2016; Cope et al., 2022). The emergence of PNNs in the CA2 appears to coincide with social recognition function during development (Diethorn and Gould, 2023a). In adulthood, CA2 PNNs are important for the ability to distinguish between novel and familiar mice; degradation of PNNs impairs this function in otherwise healthy mice (Cope et al., 2022). Conversely, inbred and genetic mouse models of social dysfunction have been associated with excessive PNNs in the CA2; some functional deficits can be restored by lowering levels of PNNs to control values (Carstens et al., 2021; Cope et al., 2022; Diethorn et al., 2026). These findings are consistent with our data showing a restoration of social recognition function in LBN males after reduction of PNNs by local infusion of the degradative enzyme chABC into the CA2 region. It should be noted that since chABC degrades the entire extracellular matrix, not just PNNs, it is possible that restorative effects on behavior are the result of its influences beyond PNNs. More targeted PNN manipulations via viral vectors that increase or decrease PNN constituents in specific neuron types would address this question. Despite this alternative interpretation, these findings suggest that the influence of LBN on circuitry underlying social recognition is not permanent and, instead, can be repaired (at least temporarily) by adult intervention. The duration of these improvements, as well as the possibility of permanently restoring function, remains unknown.

Our results suggest that the influence of LBN on CA2 PNNs in adult males may be specific to GAG chains, as WFA intensity was increased but no effects were observed with the main neuronal CSPG ACAN. WFA labeling and ACAN immunostaining often colabel PNNs in the hippocampus, but there are reports of PNNs labeled by one marker and not the other (Yamada and Jinno, 2017; Laham et al., 2022). Furthermore, other pathological states, such as neuropathic pain and Alzheimer’s disease, have been associated with changes in one of these markers and not the other (Scarlett et al., 2022; Tansley et al., 2022; de Vries et al., 2025), suggesting that maintaining an optimal balance of PNN constituents may be important for healthy function. Future studies should investigate whether enzymes associated with increased glycosylation of CSPGs are altered in adult LBN males and how these changes may affect social recognition.

PNNs appear to restrict plasticity by limiting postsynaptic sites available for synaptogenesis and/or by concentrating inhibitory neurotransmitter receptors so that inhibition is more robust (Wingert and Sorg, 2021). Previous work in other systems has shown that PNNs are sensitive to the environment (Laham and Gould, 2022) and neuronal activity, the latter of which appears to be negatively correlated with CA2 PNN intensity (Carstens et al., 2016; 2021), raising the possibility that the male LBN CA2 has higher PNNs because neuronal activity is diminished in the region. There are many ways this could occur, including by LBN affecting intrinsic properties of CA2 excitatory or inhibitory neurons, which have been shown to be sensitive to PNN degradation (Carstens et al., 2021). Another possibility is that LBN might alter developmental innervation from afferents outside of the hippocampus, the majority of which are excitatory (Diethorn and Gould, 2023a). If LBN diminishes excitatory inputs to the CA2, the corresponding decrease in activity in the postsynaptic region might contribute to excessive PNN formation. While the mechanism by which increased PNN intensity in the CA2 of LBN males impairs social recognition remains unknown, our data suggest that the effect may be reparable with interventions designed to correct PNN levels in adulthood.

### Sex differences in LBN effects on social recognition

Our results demonstrate divergent behavioral and cellular effects of LBN in males and females. In adulthood, LBN males have impaired social recognition ability, while in striking contrast, adult female LBN mice exhibit robust social recognition abilities. Although LBN impairs social recognition in adult males but not in adult females, females are also impacted by LBN, albeit differently, with higher social novelty responses compared with control-reared females. The extent to which this sex difference involves diametric changes in similar mechanisms and circuits remains unknown, as is whether it reflects vulnerability versus resilience in females. Future studies using additional behavioral tasks that directly measure social motivation, novelty preference, and investigation strategy, as well as those that manipulate social behavior circuits in females, would provide a more comprehensive assessment of sex differences in LBN effects.

These findings also raise questions about whether the sex differences arise from differential developmental experience or whether they emerge over time. Regarding the former possibility, a few studies have shown that rodent mothers display differential care toward male and female pups, perhaps due to a greater need for licking and grooming to stimulate micturition in males (Maggi et al., 1986; Sakamoto et al., 2021). Thus, LBN, which is known to alter maternal behavior (Bailoo et al., 2014; Gallo et al., 2019; Baracz et al., 2020; Shupe and Clinton, 2021), may do so differently toward male and female pups, with greater long-term effects observed in males. However, it remains possible that the effects of LBN are largely similar on male and female pups but that the females recover as time passes, possibly due to changes during puberty. Indeed, a recent study showed that LBN impairs social recognition in both sexes during development but that this function recovers in females after puberty (Bapat et al., 2026), suggesting that signaling molecules present during this time in females have a restorative effect on relevant circuitry. Future studies should focus on understanding how changes in signaling molecules during the peripubertal period or differential expression of stress-related genes in females interact with circuitry underlying social recognition.

## Synthesis

Reviewing Editor: Rosemary Bagot, McGill University Decisions are customarily a result of the Reviewing Editor and the peer reviewers coming together and discussing their recommendations until a consensus is reached. When revisions are invited, a fact-based synthesis statement explaining their decision and outlining what is needed to prepare a revision will be listed below. The following reviewer(s) agreed to reveal their identity: Kevin Bath.

In the current manuscript, the authors investigate the impact of early-life adversity (ELA) in the form of limited bedding and nesting (LBN) on adult social recognition and hippocampal CA2 perineuronal nets (PNNs). The authors found that LBN selectively impaired social recognition in adult males while females exhibit preserved, and in some respects enhanced, social novelty responses. These behavioral effects are accompanied by increased WFA-labeled PNN intensity within CA2 in males, and enzymatic degradation of PNNs restored social recognition performance. Overall, the manuscript is well written, the experiments are technically sound, and the findings are novel and potentially important for understanding how ELA may alter hippocampal plasticity. The attempted rescue experiment represents a particular strength of the work. However, several aspects of the experimental design and interpretation warrant additional consideration before the conclusions can be fully supported.

Major Issues

1. Mechanistic conclusions of PNN effects. A core conclusion of the MS is that increased CA2 PNNs underlie the social recognition deficits in adulthood following ELA. While the data demonstrate an association between increased WFA labeling and impaired social recognition, and that chondroitinase treatment restores behavior, these findings do not establish that elevated PNNs are themselves the primary pathological mechanism per se. Chondroitinase treatment alters the extracellular matrix broadly and may influence multiple aspects of circuit function beyond the measured increase in WFA labeling. Thus, the present data support involvement of CA2 extracellular matrix remodeling but do not definitively demonstrate that elevated PNNs are the causal substrate underlying the behavioral deficit. Given this, it would be beneficial to moderate several statements in the discussion that imply a stronger direct causal relationship to soften to significant involvement in future social behavior.

2. The rationale for assessing the density of immature granule neurons/neurogenesis is highlighted in the introduction, however, it is unclear what these data tell us. To begin, the measurement of 3R-Tau-positive cells was performed in adult male and female mice. While it is likely that LBN has long-term effects of neurogenesis, assessment of the survival of newly-born neurons would provide greater insight into the developmental consequences of ELA. Also, measurement of dendritic architecture and more importantly, the synaptic connections to the PNNs of the CA2 region would provide more direct measures of whether ELA-induced changes in neurogenesis impact CA2 PNNs and behavior. Lastly, can the authors provide rationale for performing t-tests for males and females rather than a 2-way ANOVA? It would appear that there are male/female differences in 3R-Tau expression between males and females; could this difference contribute to the male-female differences in social discrimination?

The PNN histological analysis indicates that LBN increases WGA expression (but not ACAN levels) in the CA2 region of male but not female mice. And chABC (but not pnase) treatment reduces WFA intensity and restores social behaviors. It could be noted that the chABC treatment elicited similar behavioral outcomes as was observed in female mice (i.e., increased investigation times compared to male mice). Very interesting. Overall, such results support the hypothesis that PNNs contribute to social behaviors, although additional studies would be required to fully support the statement that 'excessive CA2 PPNs inhibit social recognition in LBN males'.

3. Methodological questions to address. In the methods, the authors indicate that pups were cross-fostered prior to random assignment of dams to the control or LBN conditions. I'm not sure if this is a strength or a potential concern with regard to the MS. Cross-fostering can constitute an independent developmental manipulation capable of altering maternal behavior, pup stress, and developmental outcomes. Because altered maternal care is discussed extensively as a potential mechanism underlying the observed behavioral phenotype, additional justification of the cross-fostering procedure and discussion of its potential influence would strengthen the manuscript. It does not dampen significantly the additive impact of LBN on the observed outcomes, but is an important concern for groups that do not engage in cross-fostering using this same paradigm (e.g. this may represent a double hit of ELA). It would be useful to clarify whether all litters underwent cross-fostering, whether pup rejection or mortality occurred or was increased relative to non-cross-fostered litters, and whether maternal behavior differed following this manipulation independent of LBN.

4. Questions about interpretation of the female behavioral phenotype. In the MS, the authors interpret the absence of impaired social recognition in females as evidence of resilience or protection from the effects of ELA. However, the behavioral findings demonstrate not only preserved discrimination but also increased investigation of novel conspecifics. This pattern could reflect altered social motivation, novelty preference, or exploratory drive rather than true resilience of the underlying circuitry. Without additional behavioral measures capable of dissociating these possibilities. The discussion should avoid equating preserved discrimination performance with complete protection from ELA. A more nuanced interpretation acknowledging alternative explanations would be beneficial.

5. Impacts of estrous monitoring on outcomes and sex disparities. The authors indicate that female mice underwent two weeks of daily vaginal cytology prior to behavioral testing to determine estrous stage. While it is admirable that the authors tracked estrous status, the repeated handling and vaginal sampling represents an additional chronic manipulation that was not experienced by males and could itself influence stress responsiveness or adult social behavior (e.g. willingness to engage in social approach or diminished handling stress in females during social testing due to higher levels of handling prior to testing). The absence of estrous effects on discrimination does not necessarily exclude the possibility that repeated sampling altered baseline social investigation. This limitation should be acknowledged in the Discussion when interpreting the apparent sex differences observed in adulthood.

6. Other measures of social recognition? The authors rely on the direct social interaction task (DSIT) to support alterations in social recognition. While this assay is appropriate for some features of social recognition, it is difficult to determine whether the observed phenotype reflects impaired social memory specifically or changes in other behavioral processes such as novelty preference, exploratory behavior, or investigation strategy. Although total investigation times were unchanged, providing some reassurance that overall social motivation was preserved, additional discussion acknowledging the limitations of relying on a single behavioral assay would strengthen the manuscript. Moreover, the authors highlight the varied results from prior studies that have examined the effects of the limited bedding and nesting (LBN) paradigm on social behaviors. Given the heterogeneity of prior results, perhaps LBN/ELA affects some but not all social behaviors and those behaviors that are affected are also associated with structural changes in PPNs. Some discussion of this potential limitation should be added.

Minor Issues

- The male-female comparisons in Figure 1 are striking and are among the more interesting/important observations in the manuscript, but these data are somewhat undersold. This seems like a missed opportunity. Males and females were analyzed independently because of known baseline differences in social investigation. While this is reasonable, the manuscript repeatedly refers to "sex differences" without statistically testing for sex-by-treatment interactions. Either direct comparisons should be made or the wording should be modified to reflect that comparisons between sexes are based on separate analyses rather than direct statistical interaction tests and the use of the term sex differences should be used with caution.

- It would be helpful if the authors could clarify how n sizes were calculated. Many investigators will perform studies in one male and one female pup from a given litter (n=1), versus multiple pups from a single litter.

- The top-right image in Figure 3, panel B should be labelled as LBN male.

## Author Response

Response to reviewers We thank the reviewers for the positive synthesis of our manuscript and for the constructive comments provided for our revision. Our responses to individual suggestions are described below in bold font. Changes on the revised manuscript are also indicated in bold font.

Synthesis Statement for Author (Required):

In the current manuscript, the authors investigate the impact of early-life adversity (ELA) in the form of limited bedding and nesting (LBN) on adult social recognition and hippocampal CA2 perineuronal nets (PNNs). The authors found that LBN selectively impaired social recognition in adult males while females exhibit preserved, and in some respects enhanced, social novelty responses. These behavioral effects are accompanied by increased WFA-labeled PNN intensity within CA2 in males, and enzymatic degradation of PNNs restored social recognition performance. Overall, the manuscript is well written, the experiments are technically sound, and the findings are novel and potentially important for understanding how ELA may alter hippocampal plasticity. The attempted rescue experiment represents a particular strength of the work. However, several aspects of the experimental design and interpretation warrant additional consideration before the conclusions can be fully supported.

Major Issues 1. Mechanistic conclusions of PNN effects. A core conclusion of the MS is that increased CA2 PNNs underlie the social recognition deficits in adulthood following ELA. While the data demonstrate an association between increased WFA labeling and impaired social recognition, and that chondroitinase treatment restores behavior, these findings do not establish that elevated PNNs are themselves the primary pathological mechanism per se. Chondroitinase treatment alters the extracellular matrix broadly and may influence multiple aspects of circuit function beyond the measured increase in WFA labeling. Thus, the present data support involvement of CA2 extracellular matrix remodeling but do not definitively demonstrate that elevated PNNs are the causal substrate underlying the behavioral deficit. Given this, it would be beneficial to moderate several statements in the discussion that imply a stronger direct causal relationship to soften to significant involvement in future social behavior.

We agree with this point and have altered a subheading and text in the discussion to present a more accurate interpretation of the results.

2. The rationale for assessing the density of immature granule neurons/neurogenesis is highlighted in the introduction, however, it is unclear what these data tell us. To begin, the measurement of 3R-Tau-positive cells was performed in adult male and female mice. While it is likely that LBN has long-term effects of neurogenesis, assessment of the survival of newly-born neurons would provide greater insight into the developmental consequences of ELA. Also, measurement of dendritic architecture and more importantly, the synaptic connections to the PNNs of the CA2 region would provide more direct measures of whether ELA-induced changes in neurogenesis impact CA2 PNNs and behavior. Lastly, can the authors provide rationale for performing t-tests for males and females rather than a 2-way ANOVA? It would appear that there are male/female differences in 3R-Tau expression between males and females; could this difference contribute to the male-female differences in social discrimination? We acknowledge that assessing the number of 3R-tau- labeled cells does not provide a comprehensive analysis of whether the LBN paradigm altered adult neurogenesis. In response, we have added qualifying statements in the discussion about additional measures that could be different but were not made in the present study. We agree with the reviewer about the concern regarding our statistical analysis and now present the data as a 2-way ANOVA with sex and rearing condition as variables. There is a sex difference in the number of 3R-tau labeled cells in the dentate gyrus but no effect in either sex of LBN. These revisions are indicated in the methods and the results, with a new graph provided for Figure 2.

The PNN histological analysis indicates that LBN increases WGA expression (but not ACAN levels) in the CA2 region of male but not female mice. And chABC (but not pnase) treatment reduces WFA intensity and restores social behaviors. It could be noted that the chABC treatment elicited similar behavioral outcomes as was observed in female mice (i.e., increased investigation times compared to male mice). Very interesting. Overall, such results support the hypothesis that PNNs contribute to social behaviors, although additional studies would be required to fully support the statement that 'excessive CA2 PPNs inhibit social recognition in LBN males'.

We agree with this point and have added some text in the discussion to address it.

3. Methodological questions to address. In the methods, the authors indicate that pups were cross-fostered prior to random assignment of dams to the control or LBN conditions. I'm not sure if this is a strength or a potential concern with regard to the MS. Cross-fostering can constitute an independent developmental manipulation capable of altering maternal behavior, pup stress, and developmental outcomes. Because altered maternal care is discussed extensively as a potential mechanism underlying the observed behavioral phenotype, additional justification of the cross-fostering procedure and discussion of its potential influence would strengthen the manuscript. It does not dampen significantly the additive impact of LBN on the observed outcomes, but is an important concern for groups that do not engage in cross-fostering using this same paradigm (e.g. this may represent a double hit of ELA). It would be useful to clarify whether all litters underwent cross-fostering, whether pup rejection or mortality occurred or was increased relative to non-cross-fostered litters, and whether maternal behavior differed following this manipulation independent of LBN.

All litters were cross fostered to make certain that biological littermates were not disproportionately included in either rearing group. We acknowledge that cross-fostering is an additional manipulation that has been shown to impact maternal behavior in certain species and strains, although a previous study reported no large effect of intrastrain cross fostering on C57 maternal behavior (van der Veen R, Abrous DN, de Kloet ER, Piazza PV, Koehl M. Impact of intra- and inter-strain cross-fostering on mouse maternal care. Genes Brain Behav. 2008 Mar;7(2):184-92). Furthermore, we observed no evidence of pup rejection or mortality after cross fostering. In response to this reviewer concern, we included additional details to the methods about our rationale for cross-fostering and the fact that it did not negatively impact pup health or mortality.

4. Questions about interpretation of the female behavioral phenotype. In the MS, the authors interpret the absence of impaired social recognition in females as evidence of resilience or protection from the effects of ELA. However, the behavioral findings demonstrate not only preserved discrimination but also increased investigation of novel conspecifics. This pattern could reflect altered social motivation, novelty preference, or exploratory drive rather than true resilience of the underlying circuitry. Without additional behavioral measures capable of dissociating these possibilities. The discussion should avoid equating preserved discrimination performance with complete protection from ELA. A more nuanced interpretation acknowledging alternative explanations would be beneficial.

We agree that our results indicate females are also affected by ELA, which we state in the manuscript. In the revision, we focus the sex difference on the fact that, unlike males, females are not impaired in distinguishing novel from familiar social stimuli. We agree that the female results could represent changes in social motivation, novelty preference, or exploratory drive that may mask some negative effects on underlying circuitry and have indicated this in the discussion.

5. Impacts of estrous monitoring on outcomes and sex disparities. The authors indicate that female mice underwent two weeks of daily vaginal cytology prior to behavioral testing to determine estrous stage. While it is admirable that the authors tracked estrous status, the repeated handling and vaginal sampling represents an additional chronic manipulation that was not experienced by males and could itself influence stress responsiveness or adult social behavior (e.g. willingness to engage in social approach or diminished handling stress in females during social testing due to higher levels of handling prior to testing). The absence of estrous effects on discrimination does not necessarily exclude the possibility that repeated sampling altered baseline social investigation. This limitation should be acknowledged in the Discussion when interpreting the apparent sex differences observed in adulthood.

We acknowledge that estrous cycle tracking through vaginal samples represents an additional manipulation that was not experienced by our male mice. In response to this concern, we mention this potential caveat in the discussion.

6. Other measures of social recognition? The authors rely on the direct social interaction task (DSIT) to support alterations in social recognition. While this assay is appropriate for some features of social recognition, it is difficult to determine whether the observed phenotype reflects impaired social memory specifically or changes in other behavioral processes such as novelty preference, exploratory behavior, or investigation strategy. Although total investigation times were unchanged, providing some reassurance that overall social motivation was preserved, additional discussion acknowledging the limitations of relying on a single behavioral assay would strengthen the manuscript. Moreover, the authors highlight the varied results from prior studies that have examined the effects of the limited bedding and nesting (LBN) paradigm on social behaviors. Given the heterogeneity of prior results, perhaps LBN/ELA affects some but not all social behaviors and those behaviors that are affected are also associated with structural changes in PPNs. Some discussion of this potential limitation should be added.

We acknowledge that since our behavioral analysis focused on one social task, it is possible that other effects would be observed with adjacent behaviors. We believe the effects of LBN we observed in males are not due to differences in social motivation because not only is overall investigation time unchanged, but investigation times of novel mice are not statistically different between male control and LBN groups. In response to this concern, we provide text in the discussion addressing limitations of our analyses.

Minor Issues - The male-female comparisons in Figure 1 are striking and are among the more interesting/important observations in the manuscript, but these data are somewhat undersold. This seems like a missed opportunity. Males and females were analyzed independently because of known baseline differences in social investigation. While this is reasonable, the manuscript repeatedly refers to "sex differences" without statistically testing for sex-by-treatment interactions. Either direct comparisons should be made or the wording should be modified to reflect that comparisons between sexes are based on separate analyses rather than direct statistical interaction tests and the use of the term sex differences should be used with caution.

In response to this concern, the data were reanalyzed using sex as a variable. The sex differences mentioned in the manuscript are supported by these new statistical analyses by showing significant interactions (sex by rearing condition) and significant post hoc comparisons. These changes are now indicated in the methods and results, as well as with new graphs in Figure 1. To be consistent with the analysis changes we made to the behavior and 3R-Tau data, we reanalyzed the PNN data across sexes and changed the graphs in Figure 3 accordingly. In each case, the new analyses supported our original claims.

- It would be helpful if the authors could clarify how n sizes were calculated. Many investigators will perform studies in one male and one female pup from a given litter (n=1), versus multiple pups from a single litter.

The n size represents the number of mice, not the number of male-female pairs. This is now clarified in methods section.

- The top-right image in Figure 3, panel B should be labelled as LBN male.

The image has been relabeled.
